# An improved method in fabrication of smart dual-responsive nanogels for controlled release of doxorubicin and curcumin in HT-29 colon cancer cells

**DOI:** 10.1186/s12951-020-00764-6

**Published:** 2021-01-09

**Authors:** Fatemeh Abedi, Soodabeh Davaran, Malak Hekmati, Abolfazl Akbarzadeh, Behzad Baradaran, Sevil Vaghefi Moghaddam

**Affiliations:** 1 Department of Organic Chemistry, Faculty of Pharmaceutical Chemistry, Tehran Medical Sciences, Islamic Azad University, Tehran, Iran; 2 Drug Applied Research Center, Tabriz University of Medical Sciences, Tabriz, Iran; 3 Department of Medicinal Chemistry, Faculty of Pharmacy, Tabriz University of Medical Science, Tabriz, Iran; 4 Department of Medical Nanotechnology, Faculty of Advanced Medical Sciences, Tabriz University of Medical Sciences, Tabriz, Iran; 5 Universal Scientific Education and Research Network (USERN), Tabriz, Iran; 6 Immunology Research Center, Tabriz University of Medical Sciences, Tabriz, Iran

**Keywords:** pH/thermo-responsive, Nanogels, Dual-drug delivery, Controlled release, Combination therapy

## Abstract

The combination therapy which has been proposed as the strategy for the cancer treatment could achieve a synergistic effect for cancer therapies and reduce the dosage of the applied drugs. On account of the the unique properties as the high absorbed water content, biocompatibility, and flexibility, the targeting nanogels have been considred as a suitable platform. Herein, a non-toxic pH/thermo-responsive hydrogel P(NIPAAm-co-DMAEMA) was synthesized and characterized through the free-radical polymerization and expanded upon an easy process for the preparation of the smart responsive nanogels; that is, the nanogels were used for the efficient and controlled delivery of the anti-cancer drug doxorubicin (DOX) and chemosensitizer curcumin (CUR) simultaneously like a promising strategy for the cancer treatment. The size of the nanogels, which were made, was about 70 nm which is relatively optimal for the enhanced permeability and retention (EPR) effects. The DOX and CUR co-loaded nanocarriers were prepared by the high encapsulation efficiency (EE). It is important to mention that the controlled drug release behavior of the nanocarriers was also investigated. An enhanced ability of DOX and CUR-loaded nanoformulation to induce the cell apoptosis in the HT-29 colon cancer cells which represented the greater antitumor efficacy than the single-drug formulations or free drugs was resulted through the In vitro cytotoxicity. Overall, according to the data, the simultaneous delivery of the dual drugs through the fabricated nanogels could synergistically potentiate the antitumor effects on the colon cancer (CC). 
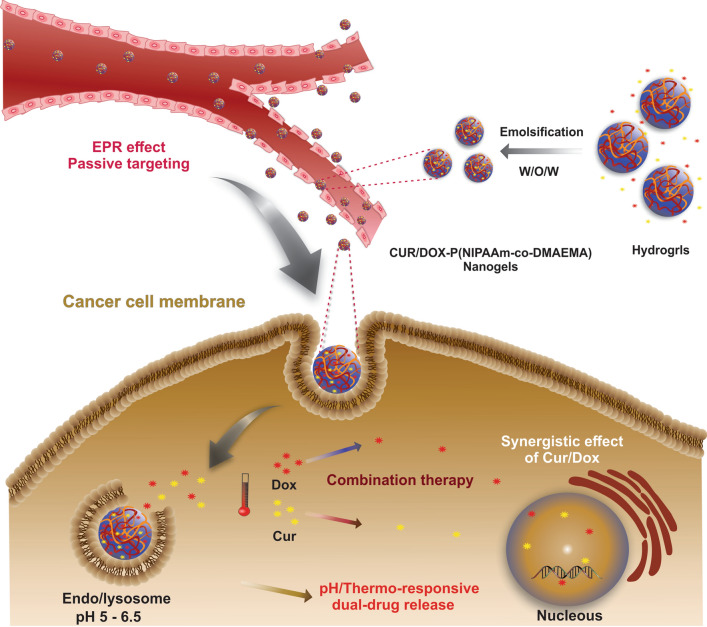

## Introduction

Cancer including the uncontrolled cell multiplication which aggressively metastasis on other parts of the body is considered a prominent cause of the death worldwide and is the generalized term for a class of the widespread diseases [1]. Although overwhelming researches have been done to stop cancer during the last decades, there are relatively few achievements in the field of cancer therapy. Despite some advancements in the cancer treatment, colon cancer (CC) has remained the third most common cancer recognized universally in the human beings [2]. The conventional chemotherapy has been known as a formal cancer treatment method accross the current cancer treatment methods including the surgical intervention, chemotherapy, radiotherapy, and a combination of these methods. The mechanism by which the chemotherapeutic agents induce apoptosis to the rapidly growing cancer cells is usually based on the interfering with the DNA synthesis and mitosis [4]. However, the nonselective action of the chemotherapeutic agents between cancerous and normal healthy tissues causes undesirable side effects that decrease the survival rate of patients. Moreover, due to the poor bioavailability of these agents, high doses are required, which leads to enhanced toxicity to the normal cells and multiple drug resistance (MDR). Therefore, the use of single-drug therapy is limited due to unaccepted toxicity in high doses and developing drug resistance [3]. Multi-drug therapy referring to the co-administration of two or more drugs with different mechanisms of action to the tumor site could be an efficient strategy to overcome the single-drug therapy’s shortfalls [4]. In a multi-drug system, the appropriate drug combinations promote the synergistic anti-cancer response through different signaling pathways, enhance therapeutic efficacy, and prevent the drug resistance [5]. Despite the positive effects of the multi-drug therapy, it has not effected desirably on the cancer treatment as a result of the low bioavailability and lack of the targeted strategy which decreases therapeutic efficacy and increases the systemic toxicity. The emergence of nanotechnology which can deliver anti-cancer agents to the site of action with improved efficacy and minimum toxicity to the healthy tissues has led to the development of nanosystems [1]. A variety of systems has been investigated for the delivery of the chemotherapeutic agents including hydrogels [7], microspheres, and nanospheres [8], micelles [9, 10], and liposomes. By using the features like selective administration of the drugs to the tumor environment through the EPR effect, active cellular uptake, extended blood circulation time, and sustained drug release, the nanoscale drug carriers could promote the treatment efficacy in order to address the challenges accompanied with the conventional chemotherapeutic agents, [6, 7]. Resembling the soft tissue microenvironment of the human body, hydrogels included the three-dimensional polymeric structures which were capable to hold a large fraction of water [8, 9]. They can be designed in the form of continuous macroscopic networks, named macrohydrogels or discrete particles, named microgels (if their dimensions are above 1 µm), and nanogels (if their dimensions are in submicrometer ranges), respectively [10]. Recent studies have demonstrated that nanoscale hydrogels (nanogels) can be an ideal system for the delivery of various chemotherapeutic agents as a result of their unique properties such as excellent biocompatibility, high dispersibility in the aqueous medium, and well-designed structures [11, 12]. Also, the higher swelling capacity of the nanogels in a water medium enhanced their drug loading capacity in comparison with other nanocarriers such as polymeric micelles and liposomes. having great loading space, they enable to encapsulate not only small drug molecules but also huge biomacromolecules such as proteins, DNA, and polypeptides. The higher loading capacity of the nanogels can be ascribed to the self-assembly through the hydrophobic and electrostatic interactions, which is important for keeping the bioactivity of drug molecules and biomacromolecules [13, 14]. In contrast to the rigid nanoparticles, nanogels with a flexible and soft structure are capable of penetrating through the tumor vasculature system, while keeping the bioactivity of the protected therapeutic agents [15]. Furthermore, their flexible properties reduce the probability of their entrapment by macrophages and prolonging their circulating lifetime [16]. More importantly, compared to the other conventional carriers like liposomes and micelles, which are less stable than nanogels, it was proven that nanogels have higher cell uptake efficacy than the other nanocarriers, leading to improvements in the in vivo bioavailability and safety of the chemotherapeutic agents [17, 18]. Among the NGs, biodegradable ones have promising applications in intelligent delivery systems due to their degradability in the cellular microenvironment and adjustable physical properties. The resultant biodegraded materials have reduced in vivo toxicity compare to the nondegradable ones. Also, biodegradable NGs can be functionalized with stimuli-sensitive groups, which enable them to identify desired cells/tissue in vivo and undergo the cleavage of a certain bond triggered by a spatial stimulus, releasing therapeutic agents in a temporally specific manner to represent optimal therapeutic efficacy. Considering above, the stimuli-responsive delivery systems have attracted much attention since they can release their payload in a controllable way if they are triggered by the external stimuli (magnetic field, light, radiofrequency, …) as well as the internal stimuli (pH, temperature, redox, …) [19]. PNIPAAm is the most recognized thermosensitive polymer displaying phase separation at a lower critical solution temperature (LCST) of ∼ 32 °C in aqueous solution [20, 21]. It precipitates as the temperature is raised above its LCST at 32–33 °C, while it is highly water-soluble at low temperatures [20–23]. The narrow LCST of PNIPAAm prevents it from the potential biomedical application since it is lower than human body temperature. To adjust the LCST of PNIPAAm around the body temperature, it can polymerize with different co-monomers. The controlled release of drugs is another main issue in the stimuli-responsive delivery system that should be solved to acquire good bioavailability and therapeutic outcomes. Diffusion, degradation, and swelling are three important mechanisms of drug release. Concentration gradient and hydrolysis of protecting polymer, favor drug release from the carrier in diffusion and degradation mechanism, while drug diffusion as a result of polymer porosity increasing in release fluid, affect the controlled release of drug by the swelling mechanism. To achieve efficient drug release, it's not desirable to use the single responsive polymer due to the complex microenvironment of tumor tissue. In this regard, preparing dual sensitive polymers, capable of drug release in response to external/internal stimuli is favorable. Among them, temperature/pH-responsive hydrogels play an important role in developing intelligent polymeric nanostructures with controlled drug release [22, 24]. One of the biocompatible co-monomers that can be polymerized with PNIPAAm by free-radical polymerization, is N, N′-dimethylamino ethyl methacrylate (DMAEMA), a water-soluble cationic monomer containing pendant tertiary amine groups. Polymerization with DMAEMA import some additional properties to the hydrogels/nanogels, such as induction of drug release triggered by the acidic microenvironment of solid cancer [25, 26]. To explore the potential biomedical applications of nanogels, Duan et al. developed a thermosensitive triple-monomer constructed nanogels P(NIPAAm-DMAEMA-AA) (PNDA) and studied the cytotoxicity of DOX-loaded PNDA nanogels in A549 cells. In almost all the studies involving the combination of poly (NIPAAm) monomer with a co-monomer, N,N-methylenebisacrylamide (MBA) was commonly applied as the crosslinking agent [27]. In a study conducted by Musia et al*.* the influence of crosslinkers EGDMA, DEGDMA, and TEGDMA on PNIPAAm microsphere’s thermosensitivity and morphology were studied. The results indicated that by increasing the crosslinker’s chain length the polymeric network was loosened due to the increase in the distance between the polymer chains, which boost the swelling capacity of the polymer and increase free volume accessible to the drugs. Safajou et al. also investigate the effect of crosslinker content on the polymerization kinetics of TEGDMA crosslinked poly(methyl methacrylate) (PMMA) hollow particles by studying the pressure and temperature profiles during the reaction. They found that, the use of higher TEGDMA concentration leads to the higher polymerization rate, a decrease in the gel time, and a higher pressure at the gel point [28]. DOX is an anthracycline antibiotic that has been widely used in clinical cancer therapy [29]. While the efficacy of the DOX is only achieved at very high doses since most of the DOX eliminated from circulation due to its short half-life, the stated antitumor agent binds to DNA and activates biochemical events, causing cell apoptosis. [30]. Dose-dependent cardiac toxicity is a major adverse side effect having limited its clinical applications [31]. On the other hand, CUR is a polyphenolic bioactive compound that can be considered as a safe anticancer agent [32]. It has also many biological activities including antioxidant, anti-viral [33, 34], anti-inflammatory, and antimicrobial [35]. It can overcome multi-drug resistance by downregulation of p-glycoprotein [36], while it suffers from limitations such as low water solubility, fast metabolism, instability, and poor bioavailability [37]. The nanocarriers-based delivery systems can be a good strategy in cancer treatment in order to tackle the problems associated with the DOX and the polyphenol CUR in combination therapy. Utilizing the DOX/CUR nanoformulations in cancer therapy can develop sustain drug release, increase the bioavailability of drugs, and reduce the required drug doses. In the previous study of our team, we designed a cellulose-based pH-sensitive nanocarrier and used it for co-delivery of model anti-cancer drug methotrexate (MTX) and CUR to the MCF-7 and MDA-MB-231 breast cancer cell lines. The cytotoxicity studies revealed that CUR as an adjuvant drug could synergize the therapeutic efficacy of the MTX and reduce the required doses of MTX which is a promising result to avoid cytotoxicity of the normal healthy cells [38]. By considering the advantages of multi-drug therapy using CUR as an adjuvant drug, in this work the pH/thermosensitive biocompatible hydrogel, poly (NIPAAm-co-DMAEMA), was prepared and converted to the smart nanogels for the co-delivery of DOX and CUR drugs. The drug-release behavior, intending to improve treatment efficiency was also studied. The fabricated hydrogels and nanogels were characterized in terms of the physicochemical properties, and the anti-tumor efficacy of the dual drug-loaded nanogels using HT-29 colon cancer cells. Furthermore, the apoptotic response and cell growth inhibition treated by the different drug formulations were studied through the cell cycle analysis and DAPI staining.

## Experimental

### Materials and methods

N-Isopropylacrylamide (NIPAAm), tetraethylene glycol dimethacrylate (TEGDMA), potassium persulfate(PPS), and Polyvinyl alcohol (PVA MW = 89,000) was purchased from the Sigma-Aldrich. N, N-dimethyl-aminoethyl methacrylate (DMAEMA) monomers, was purchased from the Merck (Darmstadt, Germany). Curcumin (merk, Germany) and doxorubicin hydrochloride (Sigma, USA) was used without further purification. Methanol (HPLC grade, Fisher Scientific, UK), Dimethyl sulfoxide (DMSO), and dichloromethane (DCM) were obtained from the Merck Company. Phosphate buffer saline (PBS), MTT (3-(4,5-dimethylthiazol-2-yl)-2,5-diphenyltetrazolium bromide) was purchased from the Sigma-Aldrich Company. Fetal bovine serum (FBS) and trypsin–EDTA were purchased from the Gibco (Life technologies) (Carlsbad, USA). HT-29 colon carcinoma cells were acquired from the cell bank (pasture institute Iran).

### Synthesis and characterization of P(NIPAAm-co-DMAEMA)

For synthesizing the pH and thermosensitive P(NIPAAm-co-DMAEMA), the free radical polymerization methods were utilized [20, 22]. In brief, 3800 mg NIPAAm and 533 mg DMAEMA, along with TEGDMA (2% w/w), as a crosslinker, were dissolved in 660 µl deionized water. Before the polymerization reaction, the flask containing the desired material was purged with the nitrogen to completely remove any residual oxygen. After all, the reagents were dissolved and mixed thoroughly at 70 °C for 30 min in the presence of PPS (10% w/w) to initiate the polymerization reaction. The reaction solution was continuously stirred for 12 h under the nitrogen atmosphere to generate the P(NIPAAm-co-DMAEMA). The obtained hydrogel was purified for 72 h using the dialysis membrane with MWCO of 12,000 and dialyzed toward distilled water. The external aqueous solution was removed two times a day and displaced with fresh distilled water. Finally, the purified hydrogel was frozen and lyophilized to receive the dried product and the% yield of the polymer was obtained 81%.

### Preparation of DOX/CUR-Hydrogels/Nanogels (DOX/CUR-HGs/NGs)

In this step, the DOX/CUR-hydrogels or DOX/CUR-nanogels was prepared using two different loading methods of DOX and CUR into the P(NIPAAm-co-DMAEMA). In both methods, the DOX/CUR feeding ratio 1:1 was used. These methods include:

#### Preparation of DOX/CUR-HGs

In this method, the fabrication of DOX/CUR-HGs was conducted according to the previously reported method with a little modification [38, 39]. Briefly, 2.5 ml DOX-HCl (2 mg/ml) was added to the 5 ml solution consisting 100 mg ultrasonically well-dispersed hydrogel in the distilled water and continued to stir for 24 h at room temperature in the dark. To remove physically adsorbed DOX from the surface of the hydrogels, the DOX-loaded hydrogels (DOX-HGs) were centrifuged (9000 rpm, 15 min) and washed by the distilled water [39]. The supernatant was collected and placed in the dark to measure unloaded DOX by using the calibration curve of the drug being placed in supporting information (Additional file 1: Figure S1). Owing to the poor water-solubility of the CUR, its dissolution required to be performed under the sink condition. To improve the water-solubility of the CUR, the surfactant tween 80 and the solvent Methanol (MeOH) were added to the dissolution medium (PBS) with the optimum ratio 1: 17: 83, respectively. For CUR loading, the DOX-HGs were added to 5 ml of 2 mg/ml solution of CUR in the mixture of PBS/MeOH/Tween 80. The mixture was stirred for 24 h under the dark conditions at room temperature to encapsulate the CUR within the DOX-HGs. The hydrogels were collected by centrifugation at 13,000 rpm for 10 min. To remove the physically adsorbed CUR from the surface of nanocomposite polymer, the prepared DOX/CUR-HGs were washed by the distilled water. It should be considered that the supernatant was stored in the dark to evaluate the loading content (LC) of the CUR. Finally, the obtained DOX/CUR-HGs were lyophilized and stored at 4 °C for later use [38]. The single drug-loaded hydrogel was also prepared with the same feeding ratio of DOX and CUR to compare the encapsulation efficacy (EE) of drugs in different formulations.

#### Preparation of DOX/CUR-NGs

The second approach included the fabrication of DOX and CUR-loaded nanogels (DOX/CUR-NGs) via a modified water-in-oil-in-water (W/O/W) emulsion technique Firstly, 1 ml of (2 mg/ml) DOX solution was added to the oil phase consisting of 5 mg CUR and 50 mg nanogel in 4 ml DCM/DMSO with a ratio of 1: 1, flowed by homogenizing at 7000 rpm for 3 min to form the W_1_/O emulsion. Secondly, the obtained W_1_/O emulsion was added to an aqueous solution of 50 ml polyvinyl alcohol (PVA) 0.5%, and the mixture was homogenized again at 15,000 rpm for 10 min to generate W_1_/O/W_2_ emulsion. Finally, the double emulsion was stirred at the room temperature for 5 h to evaporate the organic phase (Heidolph Instruments, Hei-VAP Series, Schwabach, Germany). The dual drug-loaded nanogels were collected through the centrifugation at 13,000 rpm for 20 min, and they were lyophilized for later use. For measuring the concentration of the encapsulated drugs by using the calibration curve of the drugs being placed in supporting the information, the supernatant was stored (Additional file 1: Figure S1).

### Encapsulation efficiency (EE) and Loading content (LC)

In the first step, the standard calibration curves of both CUR and DOX were planned (Additional file 1: Figure S1). As mentioned before, the supernatants were taken out to estimate the amount of the unloaded drugs in hydrogels and nanogels (nanocarriers) using their calibration curves that was placed in supporting information. The concentration of the unloaded drugs was obtained by replacing their absorption and determined by UV–Vis spectroscopy, in the calibration curves. The subtraction of the unloaded drug mass from the total feeding drug mass gave the loaded drug mass. The percentage of the encapsulation efficiency (EE%) and loading content (LC%) was defined by the following equation:

$${\text{EE}}\% = \frac{{{\text{Amount}}\;{\text{of}}\;{\text{loaded}}\;{\text{drug}}}}{{{\text{Total}}\;{\text{drug}}}}\; \times \;100$$

$${\text{Amount}}\;{\text{of}}\;{\text{loaded}}\;{\text{drug}}\; = \;{\text{Total}}\;{\text{drug}}\; - \;{\text{unloaded}}\;{\text{drug}}$$

$${\text{LC}}\left( \% \right) = \frac{{{\text{Mass}}\;{\text{of}}\;{\text{the}}\;{\text{loaded}}\;{\text{drug}}\;{\text{in}}\;{\text{the}}\;{\text{nano}}\;{\text{carrier}}}}{{{\text{Nano}}\;{\text{carrier}}\;{\text{mass}}}} \times 100$$

### In vitro release study of drugs

In vitro release studies of drugs from the nanocarriers were carried out using the sample and separate method (SS) [40]. The release study of drugs from the nanocarriers was evaluated in the sink conditions, (83% PBS, 1% tween 80, and 16% methanol) at two pH values (7.4 and 5.8) and two temperatures (37 °C and 40 °C). In this procedure, 5 mg nanocarriers were dispersed in 2 ml release medium and placed into the incubator shaker that provided continuous rotaition. At the fixed regular time intervals, 1 ml of release solution was withdrawn from the release media and centrifuged at 12,000 rpm for 5 min. The equivalent fresh buffer solution was added to the media to maintain the sink condition during the experiment. The drugs amount released from the nanocarriers were detected by the UV–Vis spectrophotometer at the maximum wavelength (λ _max_) of drugs. The drug concentration in several samples was defined in triplicate. The calculation of the released drug percentage from nanocarriers was done by the following equation:

$${\text{M}}_{{\text{i}}} = \frac{{{\text{c}}_{{\text{i}}} {\text{v}}_{{\text{t}}} + \sum {{\text{c}}_{{{\text{i}} - 1}} } {\text{v}}_{{\text{i}}} }}{{\text{t}}} \times 100$$

where, M_i_ is cumulative release percentage, C_i_ shows the concentration of drug in the released solution at the time (i), V_t_ presents the total volume of release solution, V_i_ is the sample volume, and t the concentration of the total drug (µg/ml).

### Cell culture and evaluation of cytotoxicity

The human colorectal adenocarcinoma cell line (HT-29) was obtained from the National Cell Bank of Iran and cultured in RPMI 1640 medium perfected by antibiotics and FBS in the 25 cm^2^ culture flask. Cells were incubated for 24 h at 37 °C in damped air containing 5% CO_2_. When the cells population attained 70% confluency, Trypsin–EDTA was added to the flask and placed for 5 min in the incubator to detached cells. For neutralizing the trypsin, 2 ml FBS was utilized. The harvested cells were centrifuged at 3000 rpm for 8 min. Finally, the cells with fresh culture medium were seeded in 96-well microplates with a cell density of 15 × 10^3^ cells per well and incubated for 48 h at 37 °C with 5% CO_2_. To evaluate the cytotoxicity of nanocarriers and the antitumor activity of DOX and CUR, the MTT metabolic activity assay at HT-29 cells were used. After two days of incubation, the cells were treated with different concentrations of drug formulations in sterile conditions. For this purpose, the different concentrations of free CUR and CUR-loaded hydrogels (CUR-HGs) (0.01, 0.1, 5, 15, 20, 40 µg/ml), free DOX and DOX-HGs ( 0.1, 5, 15, 20, 40, 60 µg/ml), DOX/CUR-HGs (1, 10, 50, 20, 30, 100 µg/ml) and DOX/CUR-NGs (0.75, 7.5, 15, 22.5, 37.5, 75 µg/ml) were added to the fresh cell culture medium in a 96-well plate and incubated for two days at 37 °C and 5% CO_2_. The cells were treated with different concentrations of the blank nanocarriers to evaluate the biocompatibility of the nanocarriers,. The untreated cells in the medium were also used as a control with 100% viability. In continue, the culture medium of the incubated plates was replaced by 150 µl fresh PBS followed by 50 µl MTT solution (2 mg/ml) and incubated for 4 h. After that, the culture medium was discarded, 150 µl DMSO was administered into the wells, and placed for 20 min in the incubator. Finally, the absorbance of the individual wells was recorded by using an assay reader (ELISA Reader, Tecan's Sunrise) at a wavelength of 570 nm. The percentage of cell viability was calculated as follows:

$${\text{Cell}}\;{\text{viability}}\left( \% \right) = \frac{{{\text{OD}}\;{\text{of}}\;{\text{the}}\;{\text{treated}}\;{\text{cells}}}}{{{\text{OD}}\;{\text{of}}\;{\text{control}}}} \times 100$$

The inhibitory concentration (IC_50_) including the concentration of drug that inhibits 50% of cell growth was calculated by using GraphPad Prism 8 (GraphPad Software, Inc., La Jolla, CA). The combination index (CI) values were calculated according to the Chou and Talalay’s equation [41]:

$${\text{CI}}_{{\text{X}}} = \frac{{{\text{D}}_{1} }}{{({\text{IC}}_{{\text{x}}} )_{1} }} + \frac{{{\text{D}}_{2} }}{{({\text{IC}}_{{\text{x}}} )_{2} }}$$

where, (IC_x_)_1_ and (IC_x_)_2_ are the IC_x_ of DOX-nanocarriers and CUR-nanocarriers, respectively. (D)_1_ and (D)_2_ are the concentration of DOX and CUR in the dual drug-nanocarriers at the IC_x_ value.

### DAPI staining

To access the nucleus condensation of HT-29 cells treated with DOX and CUR, the formulation DAPI (4′,6-diamidino-2-phenylindole) was applied according to as follows: the cells were seeded onto the sterile 96-well microplates with the density of 15 × 10^3^ cells per well and incubated for 24 h. following the incubation, the culture medium was replaced by the fresh medium containing free drugs, free and drug-nanocarriers in which their concentration was around IC_50_ and incubated again for 48 h. Afterward, the cells were washed by PBS three times, and 1 ml freshly prepared paraformaldehyde (4% v/v) was used to fix the cells. After incubation for 60 min, the cells were permeabilized by adding 60 µl of 0.1% (v/v) Triton X-100 and incubated for 10 min. Then the nuclei of the cells were stained with 1 µg/mL DAPI solution for 10 min. Finally, DNA fragmentation and condensation in apoptotic cells were assessed under a fluorescent microscope (citation5: Bio Tek-USA) at 400 × magnification, and excitation at 405 nm for DAPI [26, 42]. The images were processed using ImageJ Software [43].

### Cell cycle analysis

To assess the efficacy of different drug formulations on the cell cycle progression of HT-29 cells, flow cytometric analysis was performed. The cells were seeded in a 6-well plate at a density of 2 × 105 cells/well and incubated at 37 °C for 24 h. Then, they treated with free drugs and single/dual drug-nanocarriers at doses around their IC50 and incubated for 48 h. After incubation, the cells were trypsinized and centrifuged at 3000 rpm for 10 min. The harvested cells were washed by PBS, fixed with ethanol 75% and stored at − 20 °C. Afterward, the cells were collected by centrifugation and washed twice by PBS. Around 50 µl RNase A (10 µg/ml) was added to resuspended cells in 500 µl PBS and incubated for 30 min. Finally, the cells were collected again by centrifugation, resuspended in a solution composed of PBS, DAPI, and Triton X-100 with the ration 1000:1:1, respectively, and kept in dark for 10 min. The samples were then analyzed in terms of cell distribution in different cell cycle phases using flow cytometer MACSQ Analyzer 10 (Miltenyi Biotec, San Diego, CA) and Flow Jo V10 software. The lowest available flowrate setting was used for analysis. The data was collected using a 408 nm (violet) laser and available detector for this laser including V1 channel with 450/50 nm filter. The results were also demonstrated in the form of a histogram to determine the apoptotic phase and measure the proportion of cells in G0/G1, S, G2/M.

### Characterizations of P(NIPAAm-co-DMAEMA)

#### FT-IR spectroscopy

The chemical structure and functional groups of P(NIPAAm-co-DMAEMA) were characterized by using Fourier transform infrared (FT-IR) spectra (Tensor 270, Bruker, German). The samples were prepared in the form of KBr pellet, a method in which the samples were mixed with the dry potassium bromide (KBr) powders and compressed into the disk form. The spectra of samples were displayed in the wavenumber range of about 400 to 4000 cm^−1^ at room temperature**.**

#### ^1^H NMR spectroscopy

Proton nuclear magnetic resonance (^1^H NMR) was recorded on a Bruker AVANCE III 400 MHz (Bruker Daltonics Leipzig, Germany) spectrometer using d-dimethyl sulfoxide (DMSO-d_6_), as the solvent, and tetramethylsilane (TMS), as an internal standard (δ = 0.00). Chemical shifts (δ) were given in part per million (ppm).

#### TGA analysis

To study the thermal stability of P(NIPAAm-co-DMAEMA), thermogravimetric analysis (TGA) was conducted using the instrument Mettler Toledo TGA/SDTA 851e under N_2_ atmosphere from 25 to 600 °C at a heating rate of 10 °C min^−1^. The initial degradation temperature (T_i_) and residual mass percent were defined from the TG curve, while maximum thermal degradation temperature (T_max_) was also collected from the DTG peaks maxima.

#### Field emission scanning electronic microscopy (FESEM)

The morphological properties of the synthesized nanocarriers and drug-nanocarriers were assessed by the field emission scanning electron microscopy. For the fabricated nanogels, one drop of the dissolved nanogels were placed on the aluminum foil and let dry. For the powder sample, the hydrogels and nanogels were sputtered with gold, and they were investigated by the FESEM instrument (MIRA3 FEG-SEM, Tescan) and (Hitachi, S4160).

#### Measuring the swelling behavior of P(NIPAAm-co-DMAEMA)

The classical gravimetric method was used to keep the study of the dynamic swelling behavior and measuring the swelling ratio of the hydrogels. In order to reach the equilibrium state, the prepared hydrogel was immersed in the distilled water at different temperatures (25, 37, and 40 °C) and two pH values (7.4, 5.8) for 24 and 48 h. The dry weight of each sample was obtained after removing the excess amount of the water by filter paper followed by weighing the sample. The ratio of the solvent weight to the polymer weight in the swollen polymer is known as the equilibrium weight swelling ratio (ESR) that, is calculated according to the following equation by considering the average value of three measurements for each sample [44, 45].

$$ESR = \frac{{{\text{W}}_{{\text{t}}} - {\text{W}}_{{\text{d}}} }}{{{\text{W}}_{{\text{d}}} }}$$

where W_t_ represents the swollen weight of the sample after the predetermined times and W_d_ is the dry weight of the sample before swelling.

#### Dynamic light scattering (DLS) technique

The hydrodynamic diameter (d.nm) and zeta-potential of the hydrogel were obtained at two pH values (7.4 and 5.8) using DLS (Zetasizer Nano ZS90; Malvern Instruments, UK). The hydrogels (100 µg/mL) were dispersed in distilled water and PBS by sonication in an ice bath for 10 min.

#### Determination of lower critical solution temperature (LCST)

The amount of 100 mg P(NIPAAm-co-DMAEMA) was immersed in 5 ml distilled water to swell, then the sample was heated from 25 up to 50 °C. The obtained changes were observed via the ratio of the u.v. transmittance curve to the increased temperature in the sample.

### Statistical analysis

Statistical analyses were conducted by applying GraphPad Prism version 8 (GraphPad Software, Inc., La Jolla, CA). All tests were performed in the triplicated and represented as mean ± standard deviation (SD) for n = 3. Data were analyzed by using the one-way ANOVA analysis. The level of significance was calculated by *p*-value. *p < 0.05 is considered significant, while **p < 0.01, ***p < 0.001, and ****p < 0.0001 are considered highly significant.

## Results and discussion

### Fourier transforms infrared (FTIR) spectroscopy

**Fig. 1:**
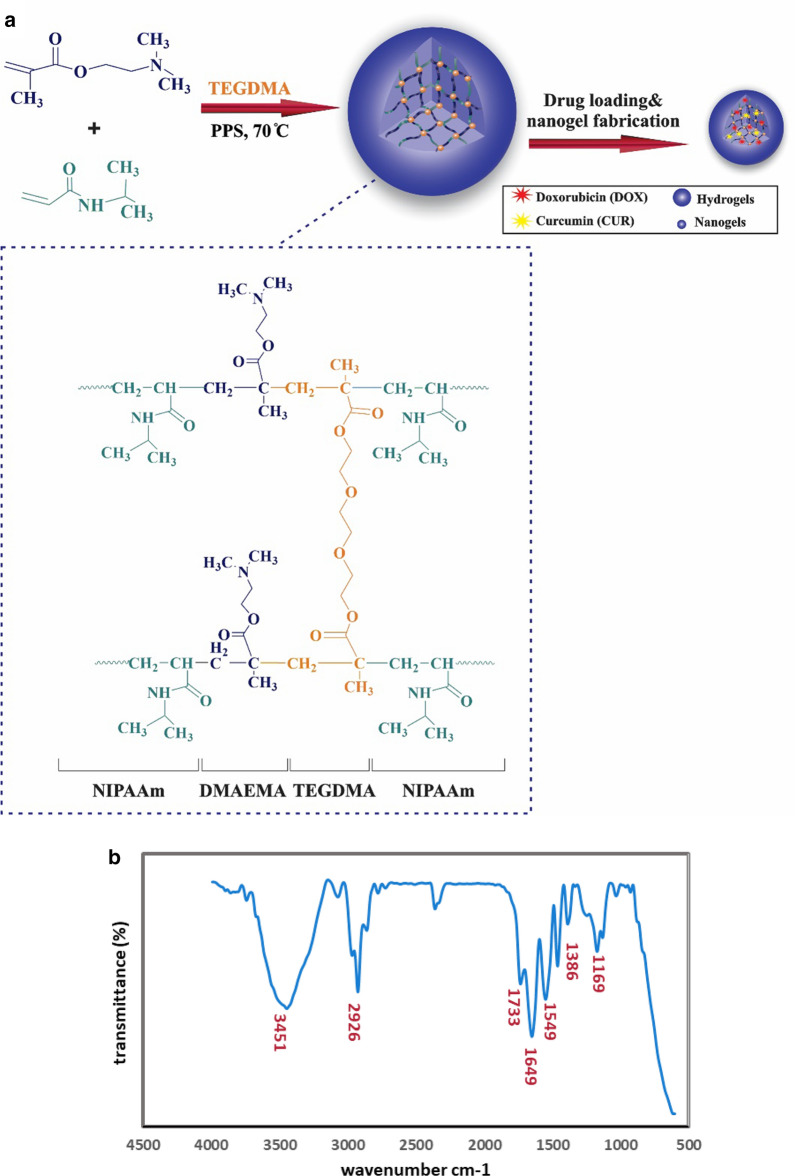

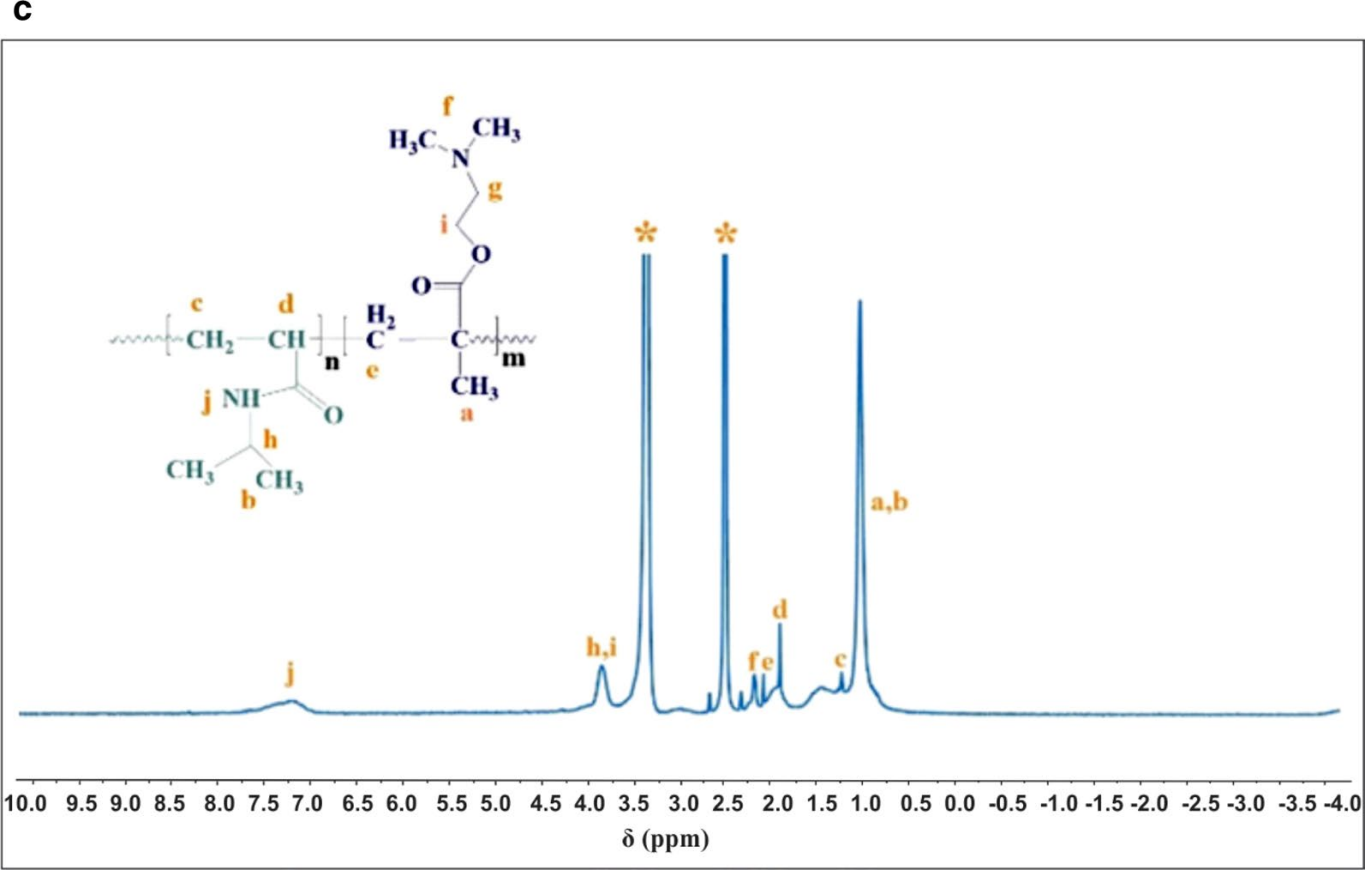
Fabrication pathway and functional groups characterization of nanocarriers. **a** synthetic steps of P(NIPAAm-co-DMAEMA) through free-radical polymerization followed by modified emulsification method. **b** FT-IR spectrum of P(NIPAAm-co-DMAEMA). **c** ^1^H NMR spectra of P(PNIPAAm-co-DMAEMA) in d_6_-DMSO using a Bruker AVANCE III 400 MHz NMR spectrometer at 298 K. Polymerization conditions were 3.8 g PNIPAAm, 0.335 g DMAEMA and TEGDMA (2% w/w) as a crosslinker at 70 °C in H_2_O for 12 h. The solvent peak was at 2.5 ppm and the water peak was at 3.35 ppm. They are represented with the asterisk symbol (*)

The co-presence of TEGDMA (crosslinker (, NIPAAm, and DMAEMA within the poly (NIPAAm-co-DMAEMA) polymer network could be characterized by using the FTIR spectrum (Fig. 1b). A signal at 1169 cm^−1^ was attributed to the stretching vibration of the C-O moiety of the DMAEMA copolymer [46]. Additionally, the strong peaks around 2926 and 1386 cm^−1^ are related to the aliphatic C-H stretching and bending mode, respectively. The broad absorption band at 1733 cm^−1^ can be attributed to the stretching vibration of esteric carbonyl (C = O) groups. Two additional peaks around 1649 cm^−1^, 1549 cm^−1^ were corresponding to the stretching vibration of C = O groups in amide functional groups and N–H bending vibration of amide groups in NIPAAm, respectively. The broad peak around 3451 cm^−1^ referred to the N–H stretching vibration of NIPAAm amide groups [47, 48].

### ^1^H NMR spectroscopy

The chemical structure of the P(NIPAAm-co-DMAEMA) was analyzed by ^1^H NMR using d_6_-DMSO as the solvent. The characteristic signals of PNIPAAm moiety were observed at 1.04 ppm (6H, (CH_3_)_2_CH), 1.46 ppm (2H, CH_2_–CH), 1.81 ppm (1H, CH–C = O), 3.84 ppm (1H, N–CH–(CH_3_)_2_), and 7.22 ppm (1H, NH-C = O), respectively. Similar analyses were reported by some related works [49, 50]. The chemical shifts related to the DMAEMA segment appeared at 0.89 ppm (3H, C-CH_3_), 2.08 ppm (2H, CH_2_-C(CH_3_)), 2.18 ppm (6H, CH_2_-N(CH_3_)_2_), 3.98 ppm (2H, CH_2_-O), respectively. The signal of the methylene group connected to the heteroatom N was masked by the solvent (DMSO) signal. The results were in accordance with the previously reported analysis of DMAEMA [51, 52].

### Temperature and pH dependence of the equilibrium swelling ratio

To investigate the effect of pH and temperature on the equilibrium of swelling ratio, a certain amount of P(NIPAAm-co-DMAEMA) hydrogel was immersed in distilled water, buffer solutions with two pH values (5.8 and 7.4), as well as different temperatures 25, 37, and 40 °C, respectively. To make PNIPAAm pH-responsive, a weak acid/base can be polymerized with it. Here, the utilized pH-responsive monomer was N, N-dimethyl-aminoethyl methacrylate (DMAEMA) with the pKa around 7.5. Upon the copolymerization of NIPAAm with DMAEMA, the polymeric network became pH-sensitive because of the protonation of the tertiary amine groups of DMAEMA at pH < pKa causing the gel swell as a result of the electrostatic repulsion and an increase in the osmotic pressure. At pH > pKa the polymer network returns to its initial state [53, 54]. It is supposed that the swelling ratio of the P(NIPAAm-co-DMAEMA) is determined by some major factors like hydrophilic/hydrophobic balance in the polymer network, electrostatic repulsion, and ionic strength. According to the Table 1, when the temperature and pH increase, the swelling ratio of the hydrogel decreases dramatically. The polymer is sensitive to the ionic strength of the environment at the low pH where the tertiary amine groups of the DMAEMA are protonated; therefore, in the distilled water with lower ionic strength, there is the highest swelling ratio [55, 56]. In a low pH solution, the NIPAAm moiety of the polymer backbone exhibited slight dehydration of the isopropyl groups leading to the disappearance of some hydrogen bonds between N–H and C = O groups and changing the chains to the extended form. The reduction in the number of hydrogen bonds, accompanied with the electrostatic repulsion of protonated amine groups of DMAEMA, caused to the swelling of hydrogel followed by increasing the possibility of the fluid exchange with the environment. The pH-dependent release of the encapsulated drugs can corollate to the higher swelling rate of the hydrogels in the acidic medium, which may accelerate endosome disruption and enhanced the cytosolic level of drugs. On account of the increase in swelling rate, known as the proton sponge effect, the acidic facilitate drug releases [57]. When the temperature increased above the LCST of the polymer, the pendant NIPAAm chains changed into the global form and representd the hydrophobic behavior. The electrostatic repulsion became the major force, and the swelling of the hydrogel increased more than the temperatures below LCST. The DLS also confirmed the above-mentioned explanation via the hydrodynamic diameter determination of the hydrogels due to the pH and temperature changes, which will further explain in "Morphological characterization" section. In contrast, at physiological pH, the pH-responsive moiety is mostly in the initial state and the electrostatic repulsion between the ammonium groups disappeared. As a result, increasing the temperature above LCST of the fabricated hydrogel, led to the shrinkage of the polymer and decrease the swelling ratio [22].

**Table 1:** Swelling behavior of synthesized P(NIPAAm-co-DMAEMA) hydrogel in different conditions

Temperature (°C)	pH	ESR
25	6.5	3.1 ± 0.36
37	7.4	0.2 ± 0.022
37	5.8	0.225 ± 0.025
40	7.4	0.025 ± 0.019
40	5.8	0.46 ± 0.13

The data were reported as the mean ± standard deviation of three independent measurements

### Thermogravimetric (TGA) analysis

**Fig. 2:**
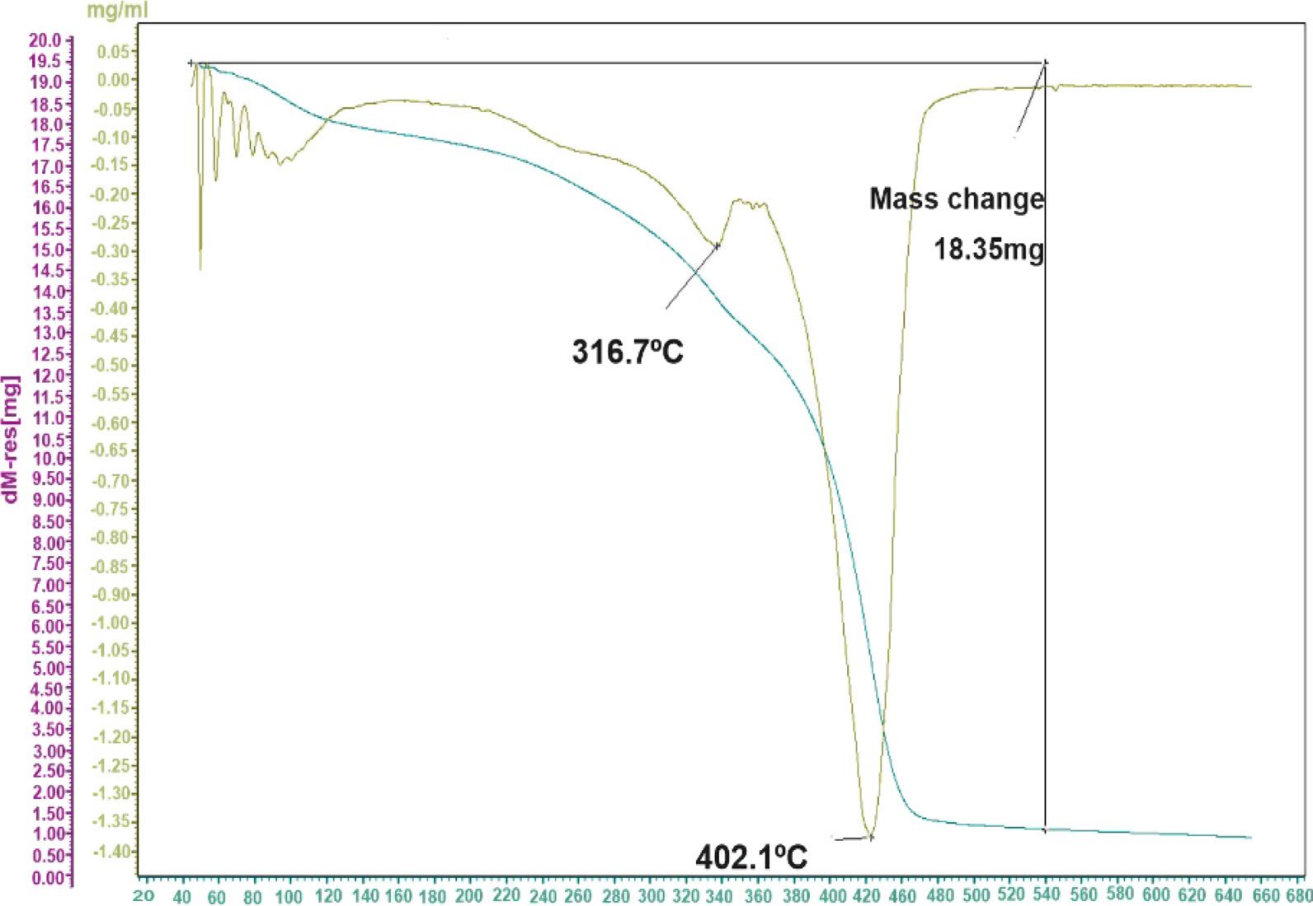
TGA and DTG thermograms displaying thermal degradation behaviors of a P(NIPAAm-co-DMAEMA)

The thermal stability and degradation behavior of the P(NIPAAm-co-DMAEMA) were investigated by TGA and DTG at 10 °C min^−1^ under the N_2_ atmosphere. The results of the TGA curve represent the amount of weight loss by increasing temperature, while the first derivative of the curve (DTG) revealed the corresponding rate of weight loss. The peak of this curve (DTG_max_) represents the degradation temperature of the polymer and can be used to compare the thermal stability of the materials. The TGA and DTG curves of the sample showed 16.5% weight loss at temperatures lower than 100 °C which was attributed to water evaporation [58]. As shown in Fig. 2, the degradation process had two maximum degradation rates around 316.17 and 402.1 °C. The lower degradation temperature referred to the thermal decomposition and dissociation of organic functional groups and the carboxyl abstraction process [58, 59], while the main degradation temperature corresponded to the decomposition temperature of P(NIPAAm-co-DMAEMA) hydrogel. Some characteristic temperatures on TGA and DTG curves were presented in Table 2. As can be seen in Fig. 2, the main degradation process occurred in the range of 280–420 °C, corresponding with about 82% weight loss and represent high thermal stability of the nanocomposite in the hyperthermia process. It was evident from the TGA curve that, total weight loss of P(NIPAAm-co-DMAEMA) is about 98%, which can be attributed to the removal of organic functional groups like the hydroxyl group and decomposition of the crosslinked conformation [20].

**Table 2:** Thermal parameters derived from TGA and DTG data of P(NIPAAm-co-DMAEMA)

Data	P (NIPAAm-co-DMAEMA)
T_i_ (°C)	280
T_f_ (°C)	420
T_m_ (°C)	316.7, 402.1
ML% (180–430)	82
ML% (23–640)	98
Residual mass% (640 °C)	2

T_i_: The temperature at initial degradation

T_m_: The temperature at the maximum degradation rate

T_f_: The temperature at final degradation

Ml: mass loss

### Morphological characterization

**Fig. 3:**
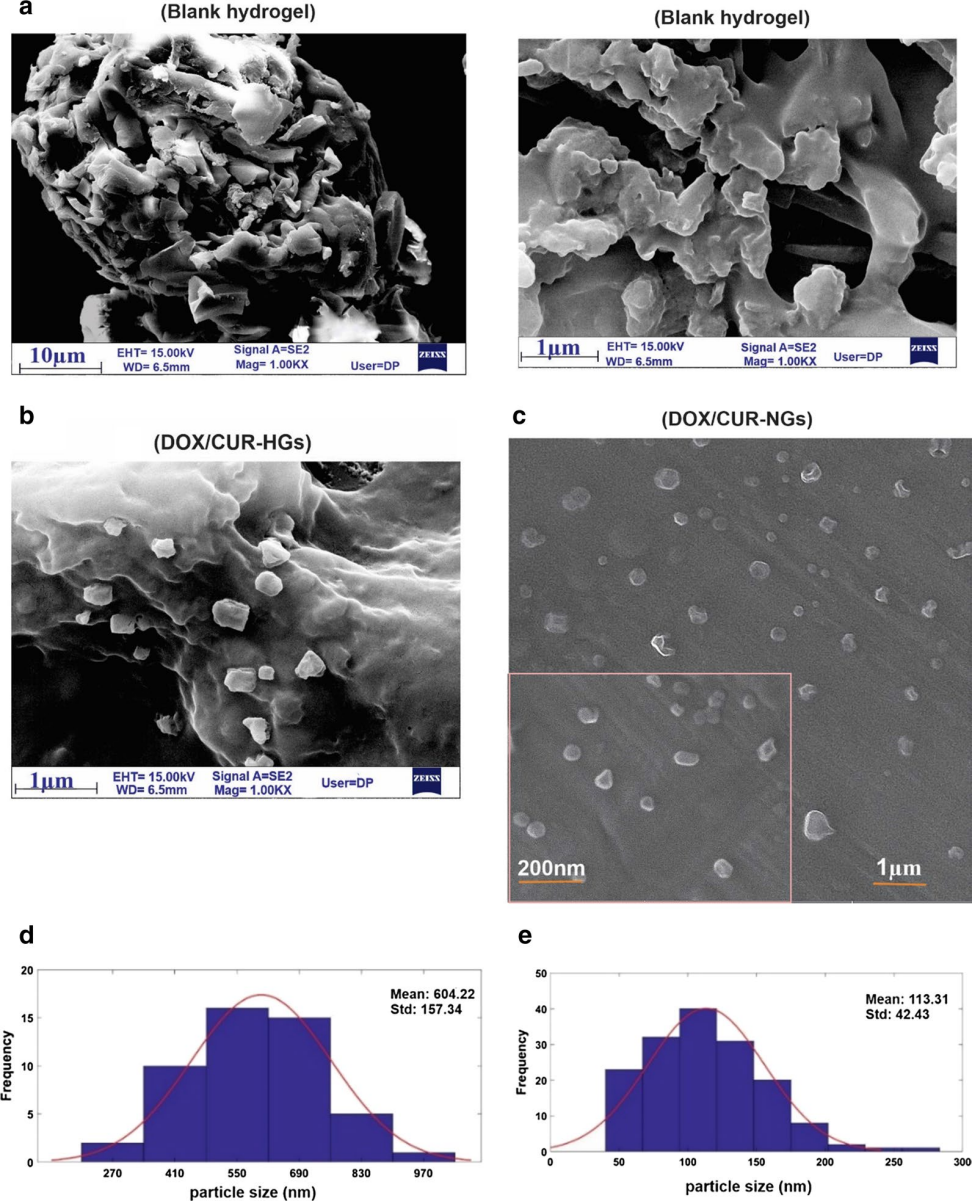
Morphological characterization of the nanocarriers. FESEM images representing the structure of (**a**) blank hydrogel, Scale bars represent 10 µm and 1 µm. **b** DOX/CUR-HGs (**c**) DOX/CUR-NGs. Scale bars represent 1 µm and 200 nm. **d** The corresponding diameter distributions of the hydrogels, **e** The corresponding diameter distributions of nanogels

To study the morphology, size, and structure of the P(NIPAAm-co-DMAEMA), FESEM was performed. The FESEM micrograph of a blank hydrogel, DOX/CUR-HGs, and DOX/CUR-NGs are presented in Fig. 3. The rigid boundaries topology and a slightly larger size in the blank hydrogel compare to the DOX/CUR-nanocarriers are shown in Fig. 3a. The results of DOX/CUR-HGs and DOX/CUR-NGs morphology assessing revealed the uniformity in the size and shape with round topology (Fig. 3b, c). In nanogels, after encapsulation of DOX and CUR by emulsion process, the size of the particles decreased, and dispersion of the particles was improved (Fig. 3c). The emulsification process is created a stable system due to the favorable contact between oil and water phases using a suitable surfactant. The function of the surfactant is to decrease the interfacial tension between water and oils, preventing the coalescence of water droplets, which finally leads to reduce the droplet size of emulsion [68, 69]. As a result, the corresponding diameter distributions of the nanogel decreased significantly compared with hydrogel. Specifically, the average diameter of hydrogels were 604.32 ± 154.34 nm (Fig. 3d), while the average diameter of the nanogels were 113.31 ± 42.43 nm (Fig. 3e).

### Evaluation of size and zeta potential by (DLS) technique

**Fig. 4:**
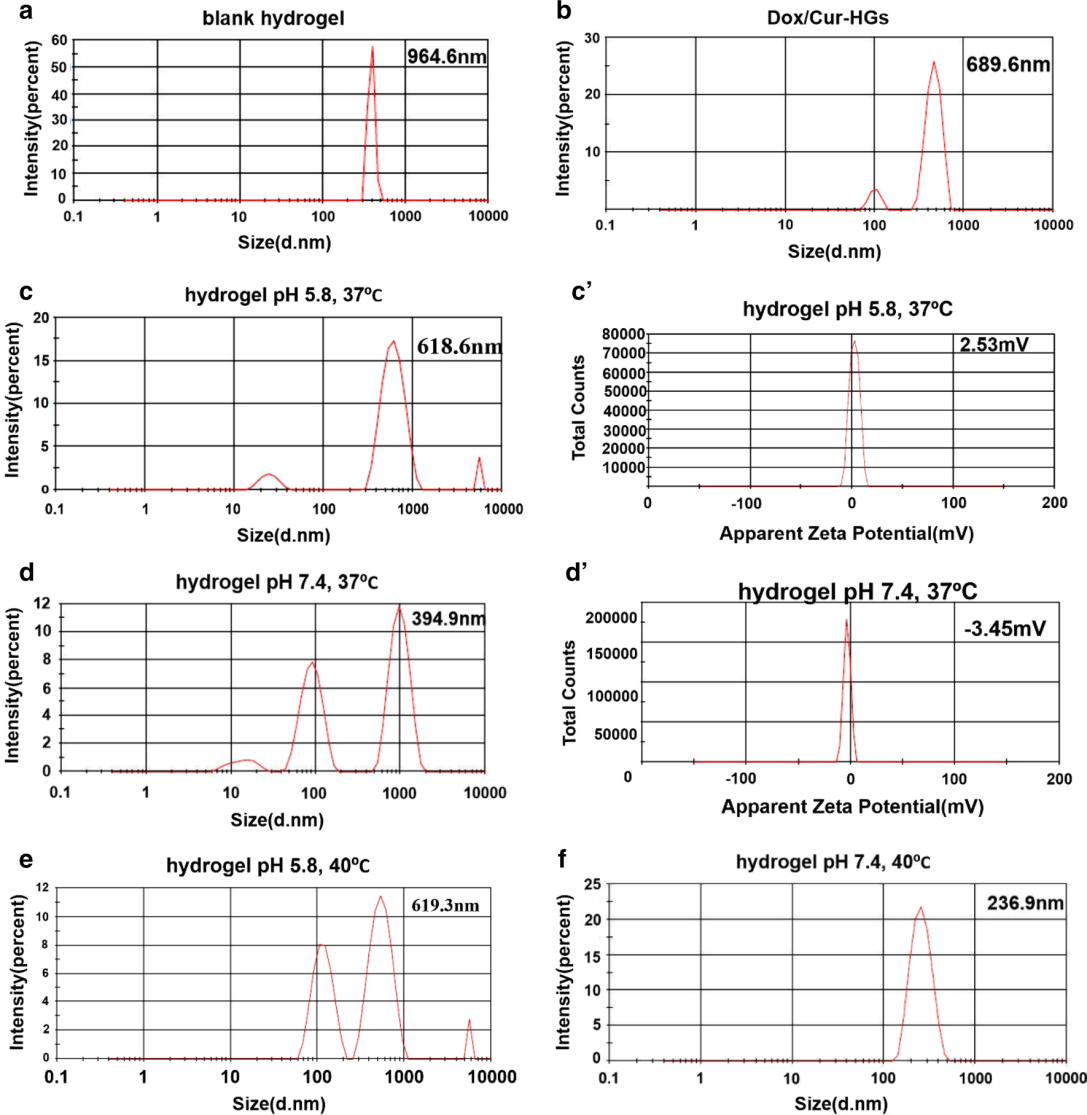
Hydrodynamic size distribution, the zeta potential of synthesized P(NIPAAm-co-DMAEMA) hydrogels in different conditions. **a** Size distribution of hydrogel in distilled water and room temperature. **b** Size distribution of dual drug-loaded hydrogels in distilled water and room temperature. Size distribution, the zeta potential of hydrogel (**c**, **c'**) in pH 5.8, 37 °C. **d**, **d'** in pH 7.4, 37 °C. The size distribution of hydrogel (**e**) in pH 7.4, 40 °C. **f** in pH 5.8, 40 °C

The DLS technique was applied to determine the particle size distribution and zeta potential for hydrogel at two pH values (7.4, 5.8) and temperatures (37 °C, 40  °C). The results are shown in Table 3. To evaluate the particle size, the blank and dual drug-loaded hydrogels was dispersed using a probe sonicator (300 w, 20 s). The formation of surface hydration layers and pseudo-clusters caused the sizes obtained by DLS in order to be slightly larger than the particle size measured by FESEM [55 Figs. 4a, b demonstrate the particle size distribution for blank hydrogel and DOX/CUR-HGs in distilled water and room temperature around 994.6 and 689.9 nm, respectively. The mean particle size distribution of the DOX/CUR-HGs is lower than the blank hydrogel probably due to the decrease in the amount of electrostatic repulsion between polymer chains. Since, in physiological pH values (drug loading conditions), electrostatic interaction occurs between functional groups in nanocarriers and drugs, which cause the copolymer chain to shrink [42]. To prove the pH-sensitivity of P(NIPAAm-co-DMAEMA) zeta potential analysis was conducted at pH 7.4 and 5.8 in 37 °C. The results are shown in Table 3. As can be seen, the amount of zeta potential and particle size for the hydrogel at pH 5.8, was 2.53 mV and 618.6 nm, respectively, while at pH 7.4 was -3.45 and 394.5. (Fig. 4c, c'). This evidence may be explained by the protonation of tertiary amine groups on the surface of PDMAEMA at lower pH values and generation of intense electrostatic repulsion, which leads to an increase in the size of particles. Whereas, with increasing pH to 7.4, the zeta potential value for the hydrogel decreased to – 3.45 mV which leads to a reduction in particle size (Fig. 4d, d') [47]. Due to the presence of DMAEMA, the copolymer becomes more hydrophilic and forms more hydrogen bonds between the polymer chains and water molecules in the physiological pH (7.4), which causes a compact hydrogel network (Fig. 4e, f) [47]. It can also be noted that the size distribution was raised at 40 °C and acidic pH to 619.3 nm, while at the same temperature and pH 7.4, it reduced to 236.9 nm. As appraised from the evidence, the results of DLS are complementary to the swelling section.

**Table 3:** Physicochemical properties of synthesized hydrogels in different conditions

Formulation	pH /Thermo	Size (nm)	Zeta potential (mV)
Hydrogels	7.4 /37 °C	394.9	− 3.45
Hydrogels	7.4 / 40 °C	236.9	–
Hydrogels	5.8 / 37 °C	618.6	2.53
Hydrogels	5.8 / 40 °C	619.3	–
Hydrogels	6.5 / r.t	994.6	–
DOX/CUR-HGs	6.5 / r.t	689.9	–

### Investigation of LCST nanocomposite and UV–Vis spectroscopy

**Fig. 5:**
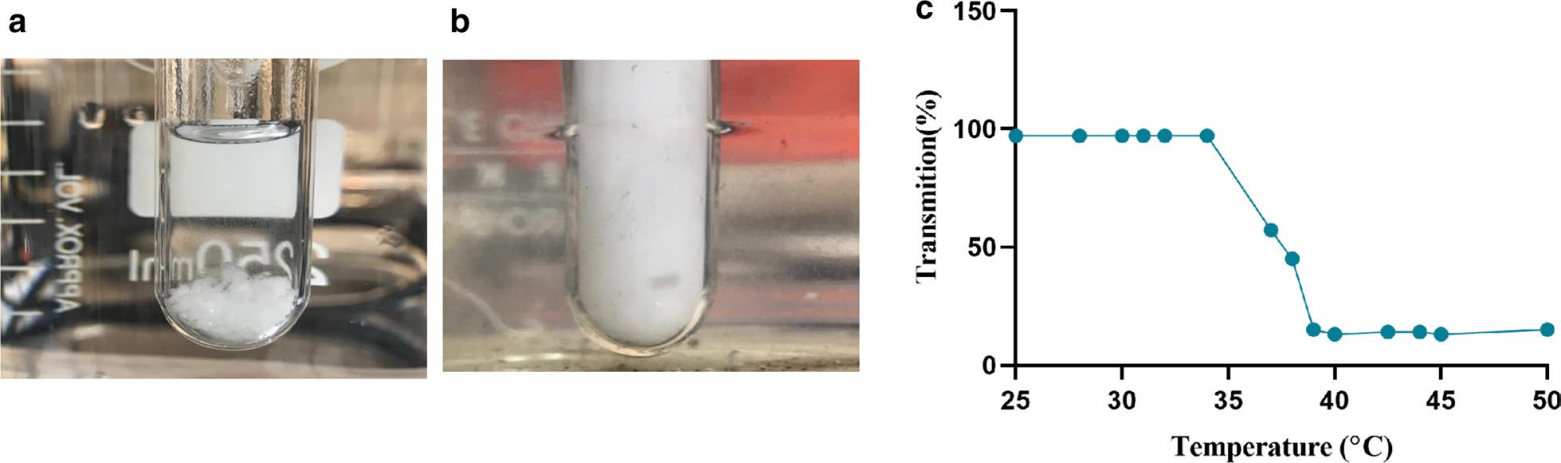
A visual illustration of P(NIPAAm-co-DMAEMA) aqueous solution and LCST determination using the UV–Vis spectrum. **a**, **b** P(NIPAAm-co-DMAEMA) aqueous solution images below and above LCST, respectively. **c** LCST determination by UV–Vis spectrum

PNIPAAm is introduced as a thermo-responsive moiety in the polymer backbone, which creates opportunities for biomedical applications [60]. A thermosensitive polymer represents significant hydration-dehydration changes in an aqueous solution near the LCST, which simultaneously undergoes a volume phase transition and the volume collapse [61]. The LCST of PNIPAAm hydrogel could be modulated by feeding the polymeric network with the DMAEMA monomer. The resulted P(NIPAAm-co-DMAEMA) has hydrophilic property due to the increasing amount of hydrogen bonds with water molecules which demand more energy to destabilize the prepared hydrogel and cause to display a higher LCST [62]. The LCST of the sample can be determined by assessing the reduction of transmittance in the UV–Vis upon heating of the sample up to 40 °C which is a rather sudden phenomenon. As depicted in Figs. 5a, b, at 25 °C, the sample is completely transparent, with a high transmittance percentage; however, at 40 °C, it becomes dim, and the amount of the transmittance percentage reduces. The amounts of the transmittance percentages were considered as a function of temperature. According to the reported researches [63–65], the LCST of the PNIPAAm is in the ranges of 32–37 °C. As can be seen in Fig. 5c, the LCST for P(NIPAAm-co-DMAEMA) was obtained in the ranges of 39–40 °C. The DLS studies further confirm the thermo-sensitivity of the hydrogel. Thus, at 25 °C, hydrogel particle size increases to 994.6 nm due to PNIPAAm branches unfolding and changing into the random coils as a result of the hydrogen bonds establishment with water molecules. When the temperature increased above the LCST (40 °C), the particle size decreased to 689.9 nm becuaes the weakening of intermolecular hydrogen bonded with a water molecule that leads to the water releases and strengthens the intramolecular hydrogen bonds. As a result, the hydrogel network precipitate as a solid gel out of a solution [66].

### Assessing the encapsulation efficacy of DOX and CUR

**Fig. 6:**
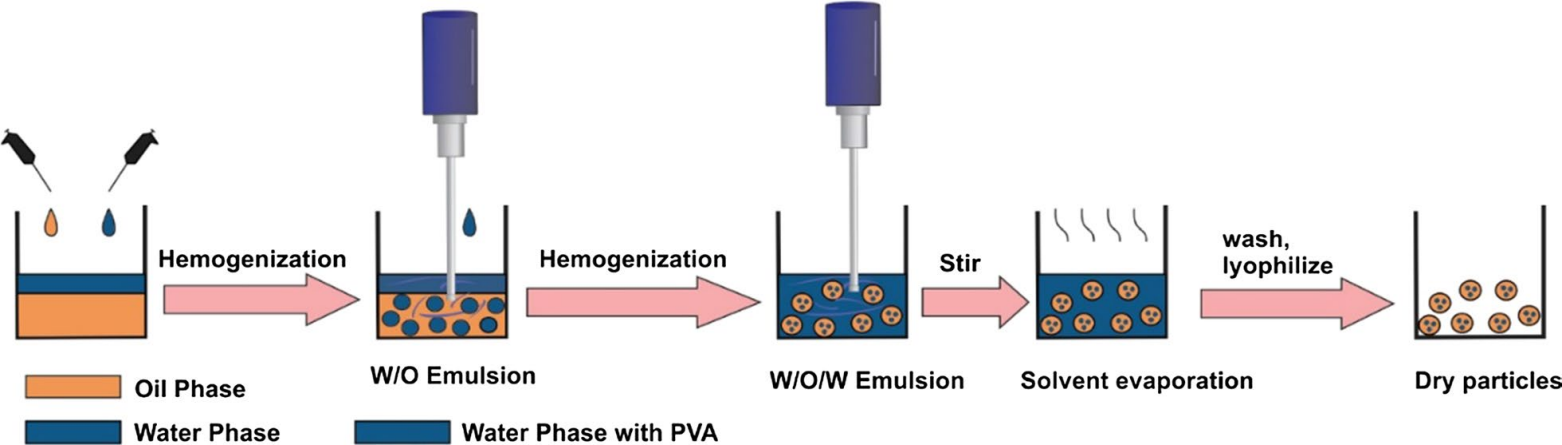
Schematic illustration of nanoparticle fabrication method. The double emulsion, solvent evaporation method was used to manufacture nanogels with encapsulated DOX and CUR drugs

In this study, both hydrophilic and hydrophobic drugs including DOX and CUR respectively, incorporated into P(NIPAAm-co-DMAEMA) by two methods. The drug loading in the prepared hydrogel happens through physical entrapment that can be related to the electrostatic and hydrophobic interactions between the polymer chains and the drug molecules [31]. In the first approach, the hydrogel is allowed to swell in the solution drugs. The swelling property allows the carrier to absorb a large amount of solution. Finally, the dual drug-loaded hydrogels were achieved after freeze-drying. In the second method that was demonstrated in Fig. 6, the hydrophilic drug (DOX) is dissolved in an aqueous phase named as an internal phase and emulsified into an oily phase that contains the polymer and hydrophobic drug (CUR). Then, the obtained emulsion is emulsified again into the aqueous solution of PVA, which was known as an external phase [67, 68]. Due to the osmotic gradient, the phenomenon of the thermodynamic driven diffusive exchange of water and oil between the internal phase and external phase happened by the surfactants at the interface of water–oil, that can lead to the production of a simple emulsion or even disappearance of the multiple globules [69]. Also, it causes the swelling or shrinkage of the inner droplets, followed by rupture of the oily layer [70, 71]. This topic effectively neutralizes the diffusive driving force for departing of hydrophilic drugs from the nanoparticle and supplies the possibility for additional loading via surface adsorption or diffusion of both hydrophilic and hydrophobic drugs into the nanoparticle[68, 70]. Multiple-emulsions are widely used as templates to prepare nanometric carriers with encapsulated anti-cancer drugs. Herein, we modified the traditional double emulsion, solvent evaporation method to encapsulate DOX and CUR in nanocarriers using multiple external water phases. (Fig. 6). After the entrapment of drugs, loading content and release behavior was investigated using a UV–Vis spectrophotometer. Standard calibration curves of both drugs at wavelength 480 nm and 420 nm for DOX and CUR, respectively, in two pH values (7.4, 5.8) were placed in supporting information, and also the Linear fitting of the standard curves for both DOX and CUR was obtained to quantify drug loading. As shown in Table 4, the amount of DOX and CUR in either single/dual drug-loaded hydrogels is comparable with DOX/CUR-NGs. The encapsulation efficiency (EE%) and loading content (LC%) for all drugs formulations are presented in Table 4.

**Table 4:** The amount of encapsulation efficacy and loading content

Formulation	^a^EE of % DOX:CUR	^b^LC% DOX:CUR
DOX-HGs	95	9.5
CUR-HGs	98	9.8
DOX/CUR-HGs	96: 98	4.8: 4.9
DOX/CUR-NGs	60: 93	3: 4.6

^a^ Encapsulation efficacy

^b^ Loading content

### In Vitro release study of drugs from the carriers

#### Drug release from DOX/CUR-HGs

**Fig. 7:**
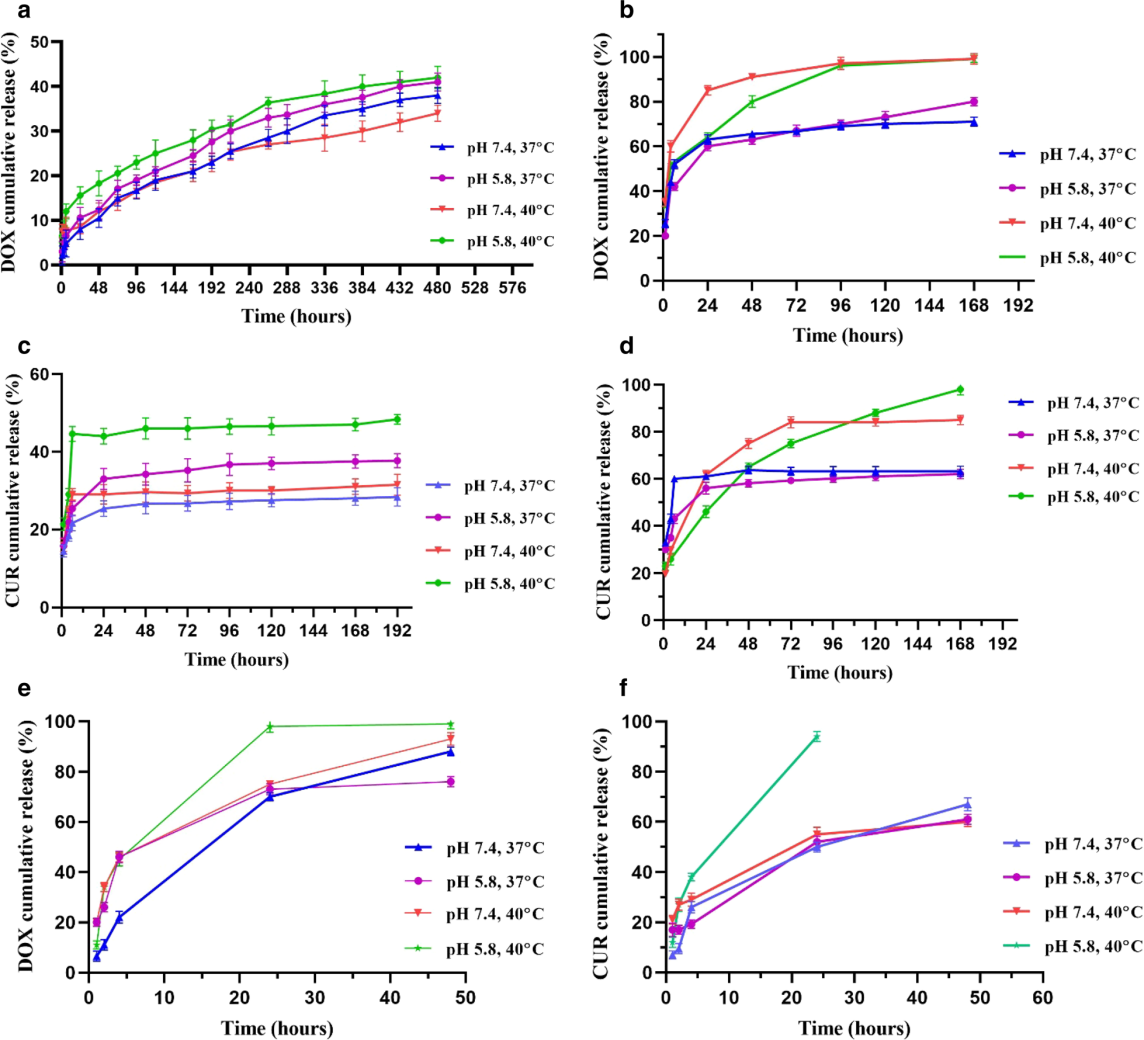
Cumulative in vitro release profiles of loaded drugs under various conditions at two pH values 7.4 and 5.8, and two temperature 37 °C and 40 °C. **a** The release profile of DOX from DOX-HGs, **b** The release profile of DOX from DOX/CUR-HGs, **c** The release profile of CUR from CUR-HGs **d** The release profile of Cur from DOX/CUR-HGs, **e**, **f** The release profile of DOX and CUR from DOX/CUR-NGs

To investigate the dual pH/thermo-responsive property of the nanocarriers, the release study of the drugs from single/dual drug-loaded hydrogels (Fig. 7a–d), and DOX/CUR-NGs (Fig. 7e, f) in the pH values of 5.8 and 7.4 at 37 °C and 40 °C, were conducted. The amount of released DOX from single drug-loaded hydrogels was quite low, so that, around 42% DOX was released at pH 5.8 and 40 °C after 20 days study, but still was significantly higher compared to pH 7.4 and 37 °C. In contrast, the release of DOX from dual drug formulation was obtained 98% at pH 5.8 and 40 °C, while by decreasing the temperature to 37 °C in the same pH condition, the release percentage decreased to 70–80% after 7 days. In general, it can be concluded that the release of DOX in the co-delivery system was higher compared to the single drug delivery system. As shown in Fig. 7c, the release of CUR from the CUR-HGs at pH 7.4 and 37 °C was slow and reached only 28%, on the contrary, its release was fast at 40 °C under acidic conditions (around 49% after 7 days). Figure 7d represents the rapid release profiles of CUR from DOX/CUR-HGs at 40 °C and lysosomal pH (pH 5.8) that reached to 80% after 48 h, while at physiological pH (pH 7.4) and 37 °C, only 60% drug released from nanocarrier. In this study, it was tried to make drug formulations pH/thermo-sensitive to reduce adverse side effects against normal cells and subsequently increase the toxic effect against malignant cells [72]. The acidic pH and high temperature cause a higher release rate of drugs as observed in the release behavior of DOX and CUR. The pH-responsive property of nanocarrier depends on the ionization degree of the drug-polymer complex on different pH conditions. In acidic pH, the carboxylate groups and amine groups of prepared hydrogels were protonated. Zeta potential study further confirmed the positive charge of the hydrogels in acidic pH. Protonation of DOX amine groups and hydrogel carboxylate groups eliminated the hydrogen bond between them and quickening DOX release in acidic conditions. Also, the protonation of CUR enolate groups (pKa_1_ 7.4) in acidic conditions promotes the release of CUR from nanocarriers [73]. The release of the drugs at 40 °C is attributed to the aggregation of PNIPAAm branches as a result of enhanced intramolecular hydrogen bonds, which leads to loosening the intermolecular hydrogen bonds with the drugs [21].

#### Drug release from DOX/CUR-NG

The release rate of drugs from DOX/CUR-NGs is faster than DOX/CUR-HGs, so the release profile examination of nanogels (Fig. 7e, f) was performed during 48 h. As depicted in Fig. 7 the releases of DOX and CUR from dual drug-loaded nanogels were more efficient than hydrogel nanocarriers, so that, at pH 5.8 and 40 °C, the cumulative release percentages for DOX and CUR in DOX/CUR-NGs were 99%, after 48 h for DOX and 24 h for CUR. However, in the same temperature and physiological pH, the cumulative release percentages of DOX and CUR were 76% and 60%, respectively. The observation of rapid drug release at pH 5.8 than pH 7.4 can be explained by the proton sponge effect of DMAEMA content of the polymer, which is extensively explained in "^1^H NMR spectroscopy" section. As reported previously, the size of particles carrying bioactive molecules such as anticancer drugs significantly influences their biopharmaceutical properties. The release profile is one of the biopharmaceutical properties in nanocarriers that size distribution in the nanometer ranges can enhance the kinetics of release through the increase in the surface area. Considering the above, it took a maximum of 48 h for DOX/CUR-NGs to release both DOX and CUR, while the release of drugs from DOX/CUR-HGs happened in a sustained manner during 168 h which led to 90% release of its payload, which further confirmed the effect of nanoparticles size distribution on the release profiles.

### In vitro cytotoxicity assay

**Fig. 8:**
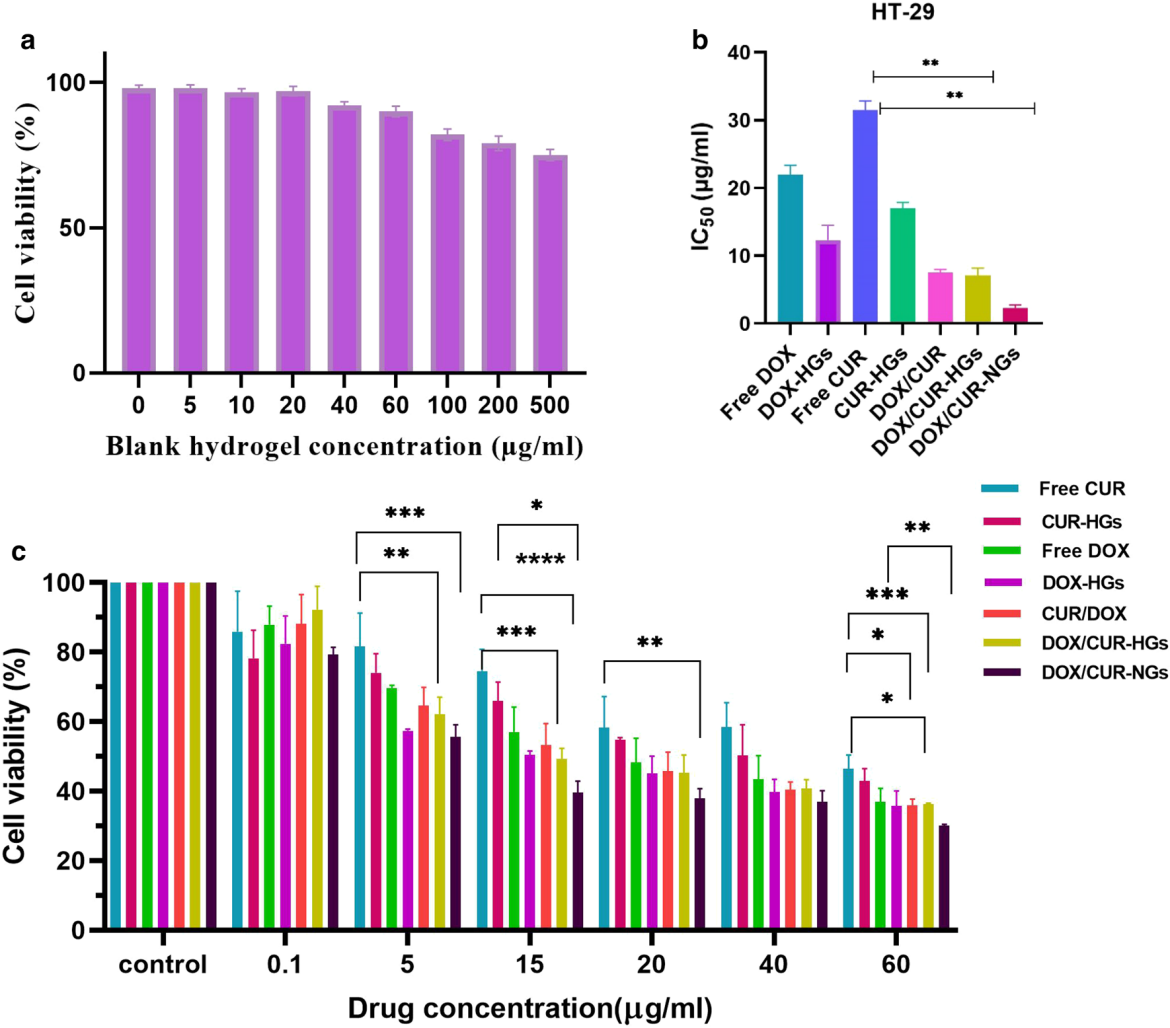
Cytotoxicity of DOX and CUR formulations in HT-29 cancer cells. **a** Cell viability of HT-29 cells after treatment with different doses of the non-drug-loaded nanocarriers. **b** The IC_50_ comparison of the different drug formulations in HT-29 cells. **c** Cell viability of HT-29 cells after being exposed to different doses of free drugs, single drug-loaded hydrogel, DOX/CUR-HGs, and DOX/CUR-NGs. Comparison among groups was conducted by one-way ANOVA, *p < 0.05, **p < 0.01, ***p < 0.001

To verify the biocompatibility and non-toxicity of blank nanocarrier and anti-tumor efficacy of DOX and CUR in different formulations, the MTT assay was conducted for 48 h in HT-29 colon cancer cells. As shown in Fig. 8a, the dose-dependent cytotoxicity of cells treated by the unloaded-hydrogel was evaluated in the concentration ranges between 5 to 500 µg/ml. The maximum viability was observed at 100 µg/ml while, by increasing the concentration up to 500 µg/ml the viability slightly decreased, suggesting the biocompatibility of the fabricated hydrogel and its potential application for drug delivery system [74]. As can be seen in Fig. 8c, the single drug-loaded nanocarriers have a more cytotoxic effect than free drugs which suggest the interference of nanocarriers in decreasing the viability of the cancer cells along with its ability to increase the drug internalization through the endocytic process. As depicted in Fig. 8b, the amount of IC_50_ for nanocarriers was much lower than free drugs and single drug-loaded nanocarriers, representing the enhanced cytotoxic effect of CUR and DOX in combination with each other. The IC_50_ amount of DOX-nanocarriers in HT-29 cells was 22.03, while when the cells were treated with CUR along with DOX, it decreased to 7.179 and 2.346 for DOX/CUR-HGs and DOX/CUR-NGs, respectively. The observed results indicate that CUR could synergize the therapeutic efficacy of anti-cancer drug DOX via induction of apoptotic cell death. To confirm the synergistic effect of the dual drug-loaded hydrogel, half-maximum inhibition concentration (IC_50_) of DOX and CUR in hydrogel along with combination index (CIx) of DOX and CUR-HGs with mass ratio 1:1 of DOX:CUR, and their cytotoxicity has compared. The combination index (CI) is a critical indicator to assess the effective interactions among multiple drugs, and the value of < 1, = 1, and > 1 suggests synergistic, additive, and antagonistic effects, respectively. The CI value 0.5 was calculated for DOX and CUR in HT-29 cells, indicating the synergistic effect of drugs. The cell viability of the samples of all drug formulations at different drug concentrations represented a completely dose-dependent pattern after treating for 48 h. CUR was applied as an active agent along with a chemotherapeutic drug DOX to persuade a synergetic effect against HT-29 cancer cells. The better cytotoxicity may be caused by the simultaneous release of the DOX and CUR from nanocarriers after internalization into the cancer cells and enhanced accumulation within the tumor site [75]. According to the results of in vitro cytotoxicity, the therapeutic efficacy of DOX and CUR in the nanogels formulation has more synergistically enhanced, that is, it represents more cytotoxicity compared to hydrogel formulation [8, 44].

### Study of induced apoptosis using DAPI staining

**Fig. 9:**
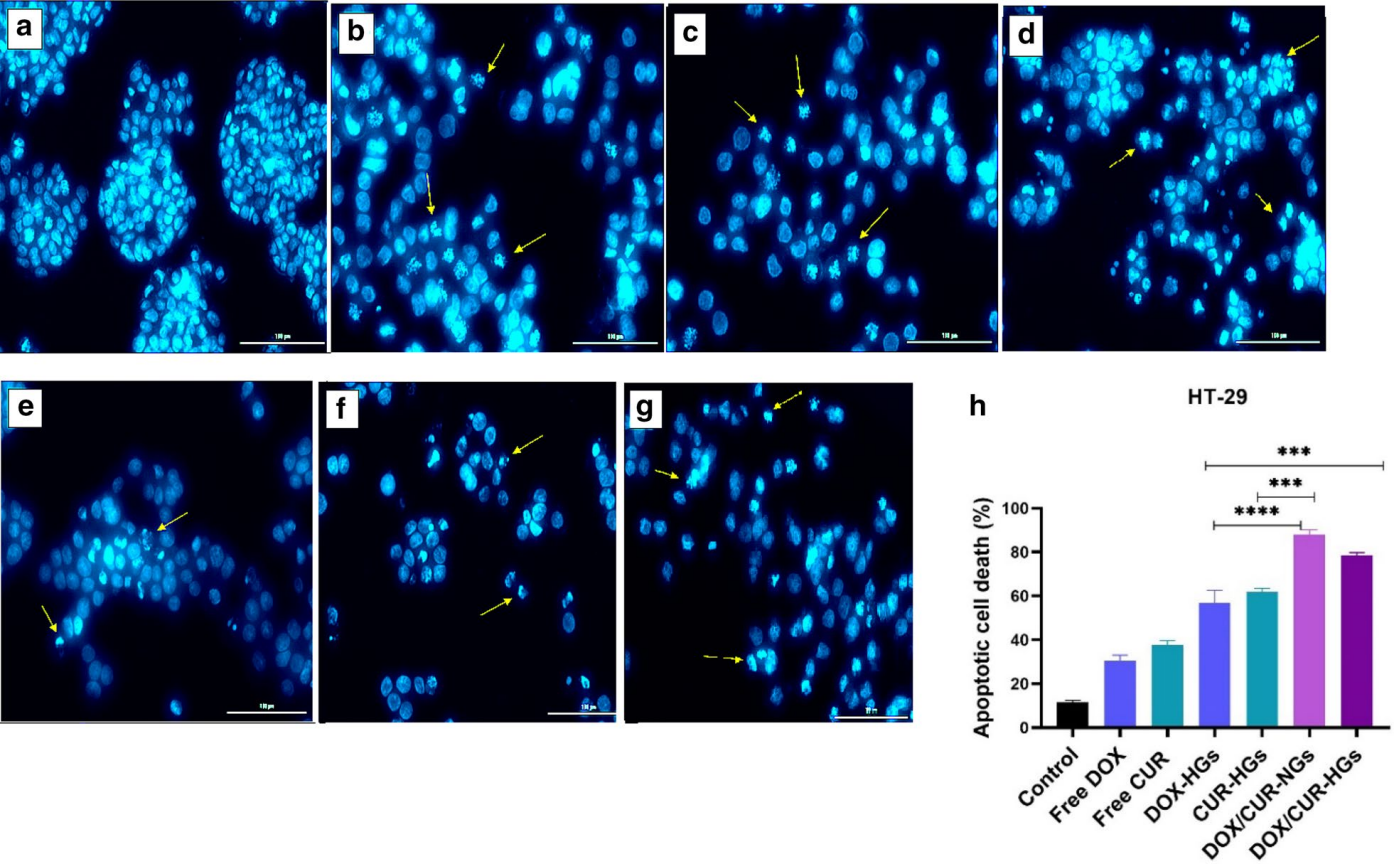
Nuclear morphology changes and apoptotic cell proportion in HT-29 cells. Fluorescence microscopy images of nuclear morphology in HT-29 cells after 48 h exposure to the **a** Untreated cells (control), **b** Free DOX, **c** Free CUR, **d** DOX-HGs, **e** CUR-HGs, **f** DOX/CUR-NGs, **g** DOX and CUR-HGs. **h** The percentages of apoptotic cell death in HT-29 cells after being exposed to free drugs, single/dual drug-loaded nanocarriers. To determine the proportion of apoptotic cells, more than 100 stained cells were counted. As depicted in the diagram, dual drug-loaded nanocarriers induced highly significant apoptosis (P < 0.001) in comparison to single drug-loaded nanocarriers. The images were processed using ImageJ Software [43]. Comparison among groups was conducted by one-way ANOVA, *p < 0.05, ** p < 0.01, *** p < 0.001

Investigation of morphological alterations induced by apoptosis in the HT-29 cells was detected by DAPI staining study. The chromatin morphological changes and the density of nuclei were observed by fluorescence microscopy after 48 h treatment. The morphological changes in cells treated with different drug formulations compared with the morphology of the untreated cells (control group). As depicted in Fig. 9a, b, the cells treated with free DOX and CUR had almost the same morphological changes, and slightly represent the sign of apoptosis. In contrast, according to Fig. 9d, e, the cells treated with single drug-loaded nanocarriers were exposed to chromatin condensation and nuclear fragmentation with more intensity. On the other hand, dual drug formulations induced more significant variations including, chromatin condensation, cell shrinkage, and strong fragmentation in the nuclei of the cells in the same dosages than free drugs and single drug-loaded nanocarriers (Fig. 9f, g). Finally, it's noteworthy to mention, DOX and CUR combination in nanocarriers with the synergistic therapeutic effect has a significant influence on the morphological changes of HT-29 cells than the other drug formulations, which can be related to the enhanced cytotoxicity in combination formulations.

### The combined effects of DOX and CUR on cell cycle distribution

**Fig. 10:**
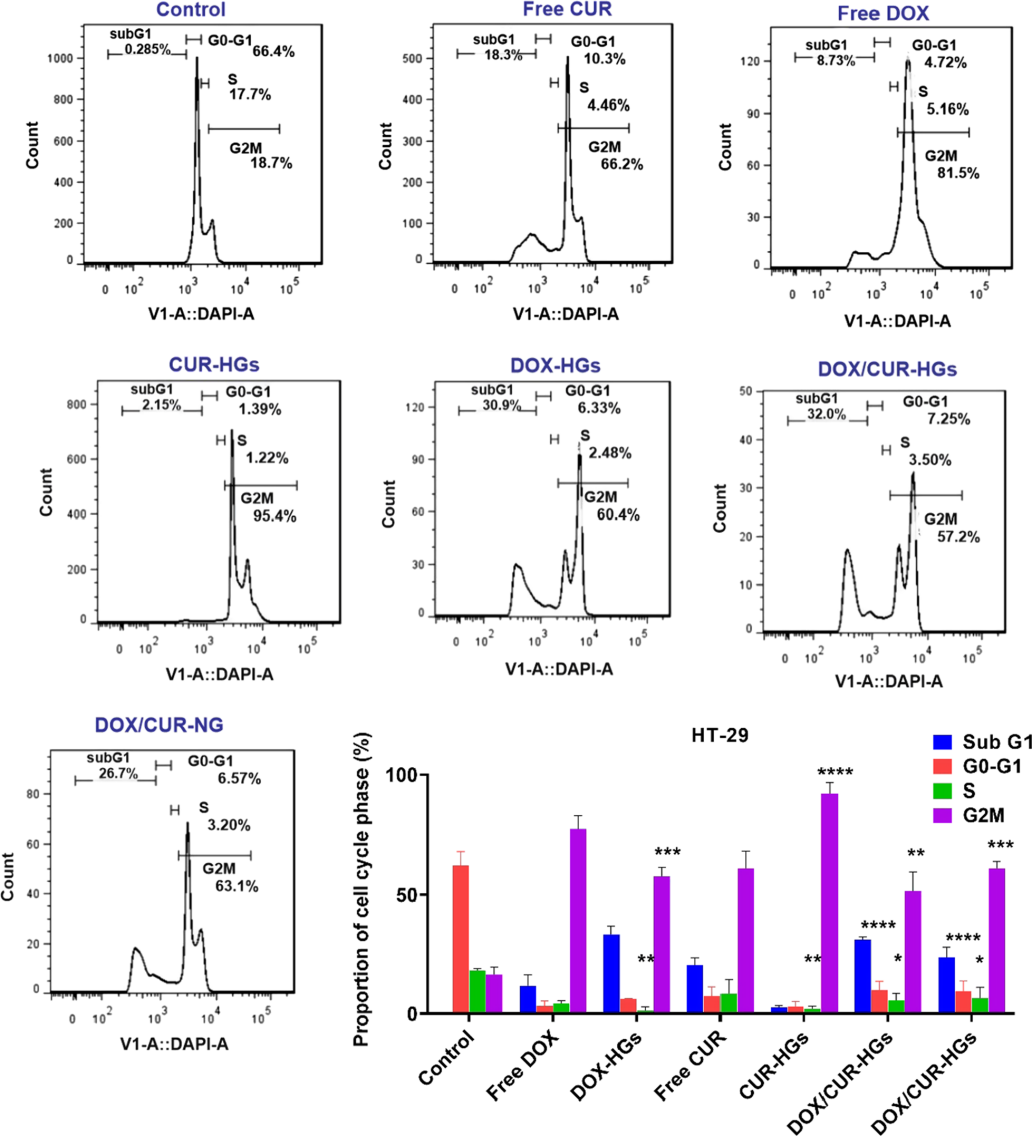
Cell cycle arrest analysis of HT-29 cells treated with different formulations of DOX and CUR. **a** Flow cytometry evaluation of DNA content in HT-29 cells after incubation with various formulations of drugs including free drugs, single/dual drug loaded-hydrogels, and nanogels for 48 h in concentrations around their IC_50_ values. **b** The proportion of cell cycle phase (%) and DNA distribution percentages in different cell cycle phases (subG1, G0-G1, S, and G2/M) for various formulations after DAPI staining in HT-29 cells. One-way ANOVA, followed by Tukey's HSD analysis, was used to determine p-values for different phases of the cell cycle. The difference was considered significant at *p < 0.05, **p < 0.01, ***p < 0.001, ****p < 0.0001

Flow cytometry applies in the cell cycle blocking studies using DNA staining indicates the percentage of cells existing in each cell cycle phase [76]. Herein, cell cycle analysis was conducted to investigate the cell cycle distribution of HT-29 cells after treated with different formulations of DOX and CUR. The drug formulations induced apoptosis via different pathways inhibit cells within the distinct phases of the cell cycle [29, 77]. Both drugs induce the accumulation of HT-29 cells in the G2/M phase [78–80]. The control of the growth and proliferation of the cancer cells in G2/M transition could be a useful checkpoint in cell cycle progression and simplify their apoptotic death [80]. As depicted in Fig. 10a, in the cells treated with free CUR and CUR-HGs formulations, while the percentage of cells in G0-G1 phase (10.3% and 1.39%, respectively) decreased in comparison with the untreated cells (66.4%), the percentage of cells in the G2/M phase (66.2% and 95.4%, respectively) increased in compared to untreated cells (18.7%). Similarly, for the cells treated with free DOX and DOX-HGs formulations, although the percentage of cells in the G0-G1 phase (4.72% and 6.33%, respectively) decreased, the percentage of cells in the G2/M phase (81.5% and 60.4%, respectively) increased in compared with untreated cells. Interestingly, the HT-29 cells treated with DOX/CUR-HGs and DOX/CUR-NGs for 48 h represent an increase in the percentage of G2/M phase (57.2% and 63.1%, respectively), and a decrease in the percentage of G0-G1 phase (7.25% and 6.57%, respectively), which is consistent with the previous studies mentioned DOX and CUR as the agents that arrest cell cycle progression in G2/M phases.

## Conclusion

In this work, a smart nanogels, based on P(NIPAAm-co-DMAEMA), were successfully developed and studied for controlled and efficient delivery of two model drugs DOX and CUR in HT-29 colon cancer cells. The resulted delivery system was characterized in terms of having the desired structure. The advantages of such nanogels systems include their simplicity of formulation, their swelling and collapse properties, and optimal loading capacity as well as the efficient release of drugs. The fabricated nanogels were used as a pH/thermo-responsive carriers that exhibited the LCST around 40 °C. It was found through the in vitro release studies that the nanocarriers released its payload in an acidic and temperature-facilitate manner so that the acidic pH and high temperature of cancer cells promoted the release of the drugs from the nanocarrier. The results of the cytotoxicity study revealed that DOX and CUR could synergistically induce apoptosis to the HT-29 colon cancer cells. Moreover, cell cycle analysis and DAPI staining studies proved the successful induction of the apoptosis by dual drug-loaded nanocarriers. In summary, the resulted smart nanogels could be served as a suitable candidate for the simultaneous delivery of hydrophilic and hydrophobic drugs, and they could achieve an efficient therapeutic activity in the combined cancer therapy.

## Supplementary Information

**Additional file1: Figure S1.** Calibration curves of Dox and Cur at pH 7.4. and Calibration curves of Dox and Cur at pH 5.4. the calibration curves of Dox and Cur at two pH values 7.4 and 5.4 were determined by measuring the absorption of Dox and Cur with known concentration using Shimatzu 1650 PC UV-Vis spectrophotometer. The absorptions as a function of Dox and Cur concentrations were recorded to construct calibration curves.

## Data Availability

The data required to reproduce these findings are available for any research.
