# Supplementary material for: An improved method in fabrication of smart dual-responsive nanogels for controlled release of doxorubicin and curcumin in HT-29 colon cancer cells
Source: J Nanobiotechnology. 2021 Jan 9;19:18. doi: 10.1186/s12951-020-00764-6 (PMC7797119; doi:10.1186/s12951-020-00764-6)
Supplement: Supplementary file 1 — Additional file1: Figure S1. Calibration curves of Dox and Cur at pH 7.4. and Calibration curves of Dox and Cur at pH 5.4. the calibration curves of Dox and Cur at two pH values 7.4 and 5.4 were determined by measuring the absorption of Dox and Cur with known concentration using Shimatzu 1650 PC UV-Vis spectrophotometer. The absorptions as a function of Dox and Cur concentrations were recorded to construct calibration curves. [file 12951_2020_764_MOESM1_ESM.docx]

*Supporting information for*

**An Improved Method in Fabrication of Smart Dual-Responsive Nanogels for Efficient Co-delivery of Doxorubicin and Curcumin in Colon Cancer Therapy**

Fatemeh Abedi^1, 2^, Soodabeh Davaran^2,3*^, Malak Hekmati^1^, Abolfazl Akbarzadeh^4,5^, Behzad Baradaran^6^, Sevil Vaghefi Moghaddam^2^

1. Department of Organic Chemistry, Faculty of Pharmaceutical Chemistry, Tehran Medical Sciences, Islamic Azad University, Tehran, Iran

2. Drug Applied Research Center, Tabriz University of Medical Sciences, Tabriz, Iran.

3. Department of Medicinal chemistry, Faculty of pharmacy, Tabriz University of Medical Science, Tabriz, Iran.

4. Department of Medical Nanotechnology, Faculty of Advanced Medical Sciences, Tabriz University of Medical Sciences, Tabriz, Iran.

5. Universal Scientific Education and Research Network (USERN), Tabriz, Iran.

6. Immunology Research Center, Tabriz University of Medical Sciences, Tabriz, Iran.

**Correspondence:** Prof. Soodabeh Davaran, Department of Medicinal chemistry, Faculty of pharmacy, Tabriz University of Medical Science, Tabriz, Iran. e-mail: [davaran@tbzmed.ac.ir](mailto:davaran@tbzmed.ac.ir)

Standard calibration curves of both drugs at a wavelength of 480 nm and 420 nm for Dox and Cur, respectively, in two pH values (7.4, 5.8) were plotted and also the Linear fitting of the standard curves both for Dox and Cur was obtained that applied for quantification of drug loading. By applying the UV-Vis spectrophotometer was estimated the amount of drug loading in the hydrogel and amount of free Dox and Cur present in the corresponding supernatants. For obtaining a concentration of unloaded drugs was replaced the resulted absorption in the calibration curves of the drugs.


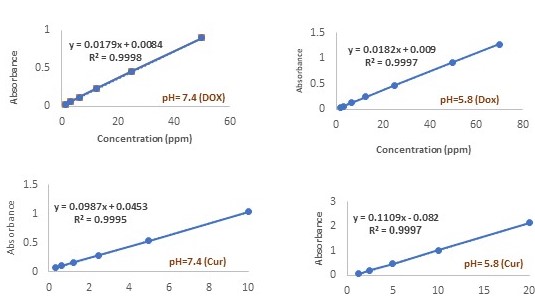


**Figure S1.** Calibration curves of Dox and Cur at pH 7.4. and Calibration curves of Dox and Cur at pH 5.4. the calibration curves of Dox and Cur at two pH values 7.4 and 5.4 were determined by measuring the absorption of Dox and Cur with known concentration using Shimatzu 1650 PC UV-Vis spectrophotometer. The absorptions as a function of Dox and Cur concentrations were recorded to construct calibration curves.
